# Child Health and Surgery: A Challenge for Future Clinical Research

**DOI:** 10.3390/children9050742

**Published:** 2022-05-18

**Authors:** Gloria Pelizzo, Valeria Calcaterra

**Affiliations:** 1 Department of Pediatric Surgery, “Vittore Buzzi” Children’s Hospital, 20157 Milano, Italy; 2 Department of Biomedical and Clinical Science “L. Sacco”, University of Milano, 20157 Milano, Italy; 3 Department of Pediatrics, “Vittore Buzzi” Children’s Hospital, 20157 Milano, Italy; valeria.calcaterra@unipv.it; 4 Department of Internal Medicine and Therapeutics, University of Pavia, 27100 Pavia, Italy

Pediatric surgical conditions cross a broad range of disease categories and includes injuries, infections, tumors, rare disease and congenital anomalies. Child Health and Surgery is based on the safeguarding of all dynamic, interactive, decision-making required to protect the becoming of the child along the different pediatric age, from neonatal period to the adolescenthood. In any condition, the pediatric healthcare promotes children’s growth, physiology, and psychosocial development and makes all efforts to minimize immediate complications and long-term sequelae due to the disease [1,2]. 

The growth interest in the treatment of a special patient, the fetus, has been largely attributed to recent advances in fetal imaging. 

Postnatal treatment of most congenital malformations prenatally detected is almost lifesaving. Fetal anomalies, previously considered fatal conditions, are now being treated using open, minimally invasive fetal surgery or percutaneous fetal technologies [3]. As the number of conditions suited to preterm babies grows, awareness and advancement of the skills are needed. A well trained neonatal surgical team and a postnatal intensive care team are considered to be essential for the best outcome of this unique population. Moreover, prenatal detection of complex malformations usually may require further interventions along the follow up of the patient through his adolescence. For this reason, pediatric surgeons are required to be skilled in a context of a multi specialistic training including urology, thoracic surgery, abdominal, plastic surgery to cope with the treatment malformations involving multisystems. Pediatric surgeons also manage emergency conditions or interventions time-critical, related to hours or days, in different stage of pediatric age. More than 50% of surgery in children is attributable to urgent indications from 0 to 16 years of age [2,4].

An emerging population in neonatal intensive care units (NICUs) and pediatric intensive care units (PICUs) is represented by chronically critically ill pediatric patients, the “fragile population” characterized by relatively higher morbidity and mortality after critical illness, that nowadays should deserve the same attention defined in adults [5]. 

Postoperative morbidity and mortality changed dramatically in the past 60 years due to the improved understanding of children physiology, the new advance in the management of critical care resorting to parenteral nutrition support, the cardiopulmonary and the dedicated pediatric anesthesia team support. Nowardays, the most recent perspectives of treatment in pediatric surgery offer a “patient taylored surgery” to optimize the prognosis and avert long-term sequelae in any condition of pediatric age.

Improvements in pediatric surgical outcomes are also attributable to refinement of the most sophisticated surgical approach such as minimally invasive technique. Minimally invasive procedures is routinely practiced in all pediatric age in thoracic and abdominal surgery; the best results recorded are in terms of pain control, reduction of postoperative pain and scars, decreasing in duration of hospital stay. 

Recently, new technique in helping guide tumor resection and the accuracy of anatomical delineation have been recently introduced in pediatric surgery for the treatment of the most recurrent malignant lesions such as neuroblastomas and Wilms’ tumors. Such surgical advances could represent a great potential to ameliorate both surgical and oncological outcomes while minimizing anesthetic time and lowering health-care costs have recently been introduced.

The surgical skilling takes on the task of defining the "patient-centric" profession based on the “taylored planning” of surgery [6]. The simulation project has been introduced in pediatric surgery clinical practice as a method to to avoid perioperative risks of error, complications, and adverse events for the best outcome. A kind of “virtual navigation” of the procedure may involves all the surgical team, anesthesiologist, nurses, surgeons for the learning of the best management of errors and the amelioration of the quality of care [7].

In this way the simulation aims to contribute to build the “specificity of care” of all professionals involved in preoperative, intraoperative and postoperative surgical management in any pediatric age and for all the specialties included in pediatric surgical filed.

Service to pediatric patients requires an understanding of their needs and expectations, and designing research that acknowledges both. The most promising models to expand infrastructure are those that include ongoing a multidisciplinary approach to optimize the result in child health and surgery in each referral Center [2,5]. The amelioration of care under multidisciplinary approach benefits on the in terms of diagnosis and management of the pediatric patients, especially in case of small babies or fragile patients. The multidisciplinary is fundamental for the therapy and involves pediatricians, obstetrics, neonatologist, radiologist, surgeons, gastroenterologist, urologists along the different phases of the pediatric age, from fetus to adolescents. Child Health and Surgery innovations in multidisciplinary pediatric management may optimize the health care in response to the special needs of the child. 

Education and continuous training of surgeons drive clinical care and strongly influence scientific discovery in pediatric field. Skills are considered essential by pediatric surgeons and represents a method for prioritizing the continuous learning in pediatric health system. It is fundamental to underline how the healthcare professionals provide care for children, and train future generations to a context of specific pediatric care. [2,5,7].

Nevertheless, short and long-term complications following all type of pediatric surgery either in neonates, children affected with rare disease or tumors, have profound and sometimes lasting effects on individual patients, families, and society. Health care in pediatrics also means to take care of the family and community life. This characteristic is unique in the health system and it deserves a dedicated consideration. Among all the Health Systems the Child Health and Surgery system development, is a special and unique model based on a team decision making which include a strong interaction with parents and caregivers [7].

Child health and surgery need to be referred to a translational and basic research program within the field of pediatric surgery as a crucial step required to adapt and offer the best treatment in respect of the child growth. As more cutting-edge therapies come into clinical practice, looking for a public opinion in terms of ethical and medical implications [2,5].

Children’s surgical care is cost-effective and children’s surgery needs to receive the much-needed specific emphasis based on the management of the complexity of care. In this regard, the world’s leading economists emphasize that Child Health and Surgery, has been ranked as one of the eight most important interventions required to improve welfare [2,5]. 

Specificity of care in pediatric field is needed to guarantee the best care and outcome in every patient and in all pediatric Centers. The pediatric surgical project should be adequately integrated into the childcare system as an indispensable addition to improve care and innovation discovery. Child Health represents a challenge of the future clinical research and should combine outcomes of clinical research methods in pediatrics with the more pragmatical data of pediatric surgery progress.
